# Book and Pamphlet Notices

**Published:** 1860-10

**Authors:** 


### Editorial.

### BOOK AND PAMPHLET NOTICES.

MEMORANDA MEDICA, OR NOTE BOOK OF MEDICAL PRINCIPLES, &c. BY HENRY HARTSHORNE, M. D., &c. J. B. Lippincott, & Co., Philadelphia: 1860.

We hail the appearance of this little volume with peculiar pleasure. It is one of the few books which approach the Baconian standard, setting forth clearly and yet with aphoristic brevity, that which is believed to be the *really known* and worthy of trust, without unnecessary comment or rhetorical display.

Years ago we uttered our earnest protest against the ponderous treatises which were, from time to time, being inflicted upon the professional public, without warrant or excuse, save the overweening pride of authorship, and weak ambition to figure upon the title page of a massive work. We begin again to believe that the earth *does* move after all. The work before us evidences a clear head, sound scholarship and large exercise of thought; nevertheless it is scarcely more bulky than the Westminster Catechism or Salem Platform. *O si sic omnia!* Herein is truth reduced to elemental propositions, readily capable of being understood, of being attacked or of being defended, without bush fighting or guerilla warfare.

The general arrangement includes, in order: Etiology, Semeiology, General Pathology, Nosology, and General Therapeutics; and to the whole is superadded what will prove of great convenience to students, a glossary. Among the special points in this treatise, we beg leave to call attention to the following. It will be recollected that Prof. Bennett asserts that inflammation is "a self-limited process which cannot be cut short or interfered with to advantage." To which Dr. Hartshorne curtly replies:

"If there be anything positive in medical experience, I believe the contrary to have been established."

Again, Dr. H. observes:

"I do not know of a single case of any kind of disease, in which the indication or object of medical treatment is to reduce the strength, or lower the vital power of the patient's system."

All of which we steadfastly believe. Again:

"It must be remembered that, at the present time, no one thinks of bleeding *for fever*, *as such*."

"Starving patients is not now thought of, unless they are fearfully plethoric. In the later stages of inflammatory disorders; in fact, *as soon as the exudation has all been thrown out*, generous diet is usually required. Some patients will never bear a purely vegetable diet under any circumstances; and *some* cases, even of inflammation, require stimulation from the first."

Another:

"Experience fully warrants the inference that mercury is a general stimulant to all those functions of organic life which are performed under innervation from the ganglia of the (so called) sympathetic system. It is probable that its action is directly *upon* those ganglia. Thus mercury tends to diffuse or equalize secretion, and the circulation of the blood, aiding, in this way, to break up local congestions and inflammations."

"The most powerful of anti-phlogistic (arterial sedative) medicine is tartar emetic."

Once more:

"Opium, always the most reliable and potent of anodyne medicines, has, in latter times, assumed a more important position as a *remedy* in the treatment of inflammatory diseases."

Which, also, notwithstanding Prof. Paine, of goodly blood-letting memory, we fervently hold to be true.

The "Concluding Maxims" of this invaluable little work, we cannot resist the temptation of reproducing in full:

"1. All pathology is but the physiology of organic perturbations.

"2. Never interfere actively in disease without a distinct object.

3. "Act only upon scientific reasons, or well defined experience.

"4. Treat the cause of disease, whenever it is possible.

"5. Watch always, and treat when requisite, the condition of the patient.

"6. Avoid, especially, routine treatment according to the *names* of diseases.

"7. Use no violence with self-limited diseases."

"I believe that a sound 'theory of medicine' may be expressed in a single paragraph, thus:

"Physiological opimism is the aggregate tendency of all the forces of the living organism, under the controlling influence of the vital force. But the best possible result in a given case may, from its conditions and circumstances, fall far short of health. Medicine, then, is to favor and supply those conditions which, under natural laws, allow or promote the best result.

"In aiming to fulfill this duty, the art of healing must always depend, in part, upon empirical observation, (which every branch of knowledge requires,) and, in part, upon inductive science. But in both alike, the physician is, or should be, *naturæ minister et interpres.*" A.

---

THE PRINCIPLES AND PRACTICE OF SURGERY; by ROBERT DRUITT, Licentiate of the Royal College of Surgeons, etc. From the Eighth Enlarged and Improved London Edition. Philadelphia: Lea and Blanchard, pp. 695, 1860. From Wm. B. Keene, Chicago.

On the appearance of the first American edition of Mr. Druitt's work, we expressed the opinion that it was an improvement on all the text books then in use. Since that time the works of Ereichsen, Fergusson, Skey, South's edition of Chelius, and others, have been so widely circulated that at the present time that opinion would require to be modified. Still, with the revision of this edition by the author, it may be said to present as fairly as could well be done in a limited space, an outline of surgical science in its present state, and will prove, as heretofore, a useful work in the hands of students and junior practitioners. Its success in England, as indicated by the number of editions it has gone through, is remarkable.

This edition has the notes of two American editors, Messrs. Flint and Sargent, so mixed up with the original, that it difficult to distinguish, without much care, what really belongs to the author himself. It is probable that the work would be quite as acceptable to the profession of this country, without the addition of the peculiar views of these gentlemen.

---

On the Theory and Practice of Midwifery. By Fleetwood Churchill, M. D., M. R. I. A.; with Additions by D. Francis Condel, M. D. A new American, from the Fourth Corrected and Enlarged English Edition. For sale by W. B. Keen, 141 Lake street.

We recognize in this book, the countenance of an old and familiar friend, in new attire, amplified and rendered more perfect by the addition of many valuable plates, by way of illustration. The rapidity with which the successive editions of this work have been exhausted, indicates the estimation in which it is held by the profession, and is a clear proof that its excellencies are fully appreciated. It is a very complete compendium of whatever has been demonstrated to be valuable in the art and science of midwifery, down to the present day. Two entirely new chapters have been appended to this edition; one treating on "Obstetric Morality," and the other on the "Qualifications and Duties of Monthly Nurses," the latter being full of instruction to the nurse, and its perusal cannot but be suggestive of useful ideas to the physician. There is no better text-book for students; or work of reference and study for the practicing physician than this. It should adorn and enrich every medical library.

---

A Treatise on Fever; its Causes, Phenomena, Treatment, &c., &c. By Rezin Thompson, M. D., Nashville, Tenn., 1860.

This is a new and considerably improved edition of a work which has attracted attention. particularly in our Southern and Southwestern States. Some fifty pages of new matter have been added to the first edition. However we might feel disposed to take exceptions to either the matter or manner of this little book, we are dissuaded from comment, to which we should otherwise be tempted, by the evidences of sincere and earnest belief, on the part of the author, that what he asserts is incontrovertibly true, and of the highest importance to be known. Such a conviction ever ought to be met, unless it results in the most patent absurdity, by generous salutation and impartial examination.

Dr. Thompson is convinced that his doctrines and treatment of fever are not surpassed in importance by any discoveries which have been made in the science of medicine. He estimates that the adoption of "A plan which would stop this outlet to human existence (febrile diseases) would save in one year, in these United States alone, at least half a million lives; a number greater than that which composed the entire belligerent forces in the Crimea. But a plan which would save half of this amount would in all fairness entitle the discoverer to be classed among the prominent benefactors of mankind. And yet this—yes, more than this—I profess to have discovered and propose to explain."

Some months ago we ventured to question the absolute certainty and reliability of sundry propositions on the fever question, by another prominent professional gentleman in Nashville, Tennessee, and although our observations were couched in the most friendly and good-natured terms, we confess that our ears have not yet recovered from the thwacking and hammering and multitudinous noises which were inflicted upon them by the indignant professor.

Our acute sensibilities will not permit us to venture the faintest shadow of a jest upon the idea that this author resides in the same city with our indignant friend above alluded to, but give a paragraph from the book before us, which explains why the coincidence is not remarkable:

"I am aware that we are all slow to acknowledge anything to be great which is the work of a cotemporary, and especially of a fellow-citizen and an equal; it requires the charm of distance, of time or space, to throw around any great discovery that prestige which commends it to our acceptance. But, of all ages of the world, this is the time; and of all places, this is the locality, in which we might most reasonably look for something great to originate; for I shall not deal much in the imaginary when I say that this is the most enlightened age of the world's history, and these United States contain more intelligence than any other country which the sun looks upon; and Tennessee is the centre State of this great Republic, and Nashville is the *sensorium commune* of Tennessee.

Dr. T. founds his system of practice upon conclusions derived from observing the peculiarities of the capillary circulations, as influenced by poisons and the nervous system. The great moving cause of the sanguineous circuit, he believes to reside in the capillaries, adopting, in the main, the views of Prof. Draper. To this, however, he superadds a *vis a fronte* which causes the return of the blood from the veins to the heart, by a *quasi* suction power, not exerted, perhaps, so much through active dilatation of the auricles as of the ventricles, and this again provoked by the capillary force of the pulmonary circulation. Mrs. Willard is complimented in this connection, which certainly is chivalrous—worthy of a Tennessean.

Disease, he concludes, "consists in debility in one or more organs, and that there can be no disease without debility, and this debility must be, of necessity, partial. A general feebleness does no more constitute disease than general power." He proceeds to illustrate by reference to the febrile results of traumatic lesion, exposure to cold, puerperal fever, and various other diseases, the effects of medicine, etc. Summing up what he thinks proved to be the essential circumstances connected with and constituting a case of fever, viz:

1st. Morbid impressions made upon the nervous centres.

2d. Weakened and vicious nervous influence sent out through the whole system.

3d. That this weakens the action of the capillaries, and causes them to dilate under the pressure of the blood, while at the same time, the sensibility of their mouths is so altered by having been supplied with morbid nervous influence, that they refuse to let the blood pass; just as the neck of the bladder, when irritated, refuses to give passage to the urine, though of a natural quality, thereby retarding the blood in the capillaries, and obstructing the circulation.

4th. That the accumulation of blood in the large vessels, from this cause, occasions the heart and arteries to be unduly excited by the stimulus of distension, thus producing the reaction which constitutes the hot stage.

5th. The pressure and tension occasioned by the engorgement of the capillaries, give rise to all the unnatural phenomena which attend a case of fever throughout its several stages, viz.: the increased, depraved, or suspended secretions, painful and unnatural sensations, inflammatory complications, etc.

6th. It is only by a partial restoration of the natural action of the capillaries that an intermission, or remission, is obtained; and only by permanently restoring this action can the disease be broken up. And lastly, if capillary action is not restored, the patient dies.

The author believes "the cause of all malarious, epidemic, and contagious fevers in organized existences, infinitesimal animalcules or sporules, which float in the atmosphere," but remarks, "whether organic or inorganic, it most certainly acts as a narcotic stimulant or nerve poison; the nature of the first symptoms indicates this most unmistakably."

"This point being settled, the field of investigation is at once open for means to neutralize the poison or counteract its effects." Hence "there are four points to be met in the treatment of every case of fever."

"1. To remove the cause.

2. To allay the nervous disturbance.

3. To restore the action of the capillaries.
4. To allay over-action and equalize the circulation.

For the first indication he proposes the oil of sassafras, as in effect generally antidotal.

For the second, valerian, especially the tincture.

For the third, piperin is "more effectual and pleasant than any other known agency."

For the fourth, sponging the surface with water of a suitable temperature; the internal use of rhubarb and super carb. soda; frictions again, externally, especially over the spine, with some kind of liniment containing both stimulant and anodyne properties. He adds:

Of course, many adjuvants have been resorted to, from time to time, to meet particular symptoms; but the above forms a general catalogue of the means by which I attempted to subdue the most potent enemy that has ever assailed our race, and with them I have succeeded, not occasionally, not generally, but uniformly and promptly. These means, though simple, as regards any unpleasant effects they can have upon the system, have proved as powerful in the contest with disease, as did the pebble which David used in his hostile meeting with the uncircumcised defier of Israel.

The particular method of compounding the several medicines used, is then given, with some detail, and sundry formulæ published. The principal one constitutes the celebrated "Fever Syrup" of the author, much in use by practitioners and planters throughout the South. [And in this connection we may notice that packages of the several medicines suitable for extemporaneous formation into the fever syrup, can be obtained by addressing either the author or Stretch & Forbes, druggists, also of Nashville, Tennessee. It is possible that Prof. Bowling, of the same place, would accommodate his friends by acting as agent for the same invaluable combination.]

In presenting the work, Dr. T. briefly considers the prominent theories which have been, from time to time, advanced on the subject of fevers, particularly the humoral, the spasmodic, the sympathetic, the phlegmasian, John Esten Cooke's, the Thompsonian, and the present; adverts to the combination of truth and error which each presents, the modes of cure suggested, and then passes to a chapter on the "Mysteries Connected with Fever," periodicity, tendency to health, curative influence over other diseases, critical days, how contagious diseases secure comparative immunity from subsequent attack; continued action of the cause, &c., &c. A very considerable degree of ingenuity is manifested in the notice of these several points, and several plausible thoughts of a novel character are brought forward.

Then follows a discussion as to the modus operandi of quinine, of topical remedies, of turpentine, in curing fever, and of mercurials in operating injuriously therein. This chapter we think the best in the work, albeit we are not yet fully wrought up to the unqualified reception of the concluding paragraph:

Sassafras does whatever the turpentine can do that is beneficial, and *never does harm*, so that it is to be preferred of the two, and, with the aid of piperin and valerian, will do pretty much all that is required to be done in typhoid or any other fever.

The remaining portion of the book is occupied in a methodical statement of the practical application of the principles and remedies previously discussed, to the treatment of particular fevers, first, however, adverting to, and in the main adopting, Prof. H. F. Campbell's views on the classification of fevers among the neuroses, claiming, incidentally, that his own doctrine with reference to the capillary system, is one of equal importance. As both the views of Prof. Campbell and his own with reference to the reciprocal relation of the nervous and capillary systems had been, long ago, clearly anticipated by another authority, to whom the professional world owe a more complete acknowledgment than yet received, we shall, for the present, pass over this portion of the subject.

Let no man fear that he may not eventually receive the encomium which is justly his due. When the intrigues of interested partisans, and the prestige of the *magnum nomen* once pass a little over, the appropriate meed will be awarded. The generalizations of Hall, Campbell, Draper, and that which this author claims with reference to the capillaries, all being but phases of another and larger view which includes and renders clear these and myriad other principles and phenomena, it is unnecessary for us here to delay to comment. The appeal lies to the coming time.

Typhoid fever Dr. T. illustrates, by various cases, treated according to his method, by himself and other practitioners. So also "bilious or miasmatic fever," pneumonia, yellow fever dysentery, cholera infantum, variola, scarlatina, and rubeola. He italicizes the rule, "*that typhoid fever treated upon the plan laid down in this book, when taken in the incipient stage, or before inflammatory complications are set up, will yield in a few days, leaving the patient but very little weakened or injured in any way.*" This position he fortifies by his own experience in large and constant practice, and by the testimony of many who have tried the same method. It will be seen that he claims not only extraordinary success in conducting typhoid fever to a successful termination, but, moreover, that he is able, when called at an early period, about invariably to abort the disease.

We must confess that, probably from dwelling in a colder clime, far from "Nashville, Tennessee," we cannot yet share this enthusiastic belief, although we think that in judicious hands the measures recommended would prove beneficial in many cases, in moderating the disease in its progress. Almost anything is an improvement over the old depleting and starvation system.

Time and space alike forbid us from further observations or comments upon this little treatise. We confess that in style and sentiment, in many parts, we find it exceedingly objectionable; nevertheless, the author gives us his positions so clearly, and appeals to experience so boldly; he challenges the profession to such simple, and, on the whole, such innoxious tests, that we are fain to say, give the matter a fair and candid trial. Buy this little book, read and laugh if you will, but when through with it, test the methods, and record the results. We have read, and admit that here and there we have laughed, and now we propose to give the method a candid and experimental investigation, and then, perchance, we may write again. A.

